# Maximising the resolving power of the scanning tunneling microscope

**DOI:** 10.1186/s40679-018-0056-7

**Published:** 2018-06-07

**Authors:** Lewys Jones, Shuqiu Wang, Xiao Hu, Shams ur Rahman, Martin R. Castell

**Affiliations:** 1 Department of Materials, University of Oxford, Parks Road, Oxford, OX1 3PH UK; 2 Present Address: School of Physics & CRANN, Trinity College Dublin, Dublin 2, Ireland; 3 Present Address: Department of Physics, COMSATS University Islamabad, Park Road, Islamabad, 45550 Pakistan

**Keywords:** STM, SPM, Multi-frame averaging, Signal to noise ratio improvement, Sub-picometre height resolution, Si(111)-(7 × 7) reconstruction, SrTiO_3_(111)-(4 × 4) reconstruction, Ti_2_O_3_ monolayer on Au(111)

## Abstract

The usual way to present images from a scanning tunneling microscope (STM) is to take multiple images of the same area, to then manually select the one that appears to be of the highest quality, and then to discard the other almost identical images. This is in contrast to most other disciplines where the signal to noise ratio (SNR) of a data set is improved by taking repeated measurements and averaging them. Data averaging can be routinely performed for 1D spectra, where their alignment is straightforward. However, for serial-acquired 2D STM images the nature and variety of image distortions can severely complicate accurate registration. Here, we demonstrate how a significant improvement in the resolving power of the STM can be achieved through automated distortion correction and multi-frame averaging (MFA) and we demonstrate the broad utility of this approach with three examples. First, we show a sixfold enhancement of the SNR of the Si(111)-(7 × 7) reconstruction. Next, we demonstrate that images with sub-picometre height precision can be routinely obtained and show this for a monolayer of Ti_2_O_3_ on Au(111). Last, we demonstrate the automated classification of the two chiral variants of the surface unit cells of the (4 × 4) reconstructed SrTiO_3_(111) surface. Our new approach to STM imaging will allow a wealth of structural and electronic information from surfaces to be extracted that was previously buried in noise.

## Background

The resolution of the scanning tunneling microscope (STM) has barely improved since its inception [1]. Only small advances have been achieved through low noise electronics, enhanced vibration damping, and low-temperature operation. These incremental gains stand in stark contrast to the advances made with the atomic force microscope (AFM). Where AFM was initially the poor cousin to the atomic resolution STM, it is now possible to take non-contact AFM (nc-AFM) images with intramolecular resolution [2]. The advantage, however, that the STM still has over nc-AFM is that the scan speed is typically around two orders of magnitude faster. In effect, this means that for the time taken to acquire one nc-AFM image it is possible to acquire around a hundred STM images. To date, this has not been viewed as a particularly significant advantage because operator practice is such that only the best one of these hundred images will be used and the others discarded. However, if all the hundred images are averaged then we would expect a tenfold improvement in the signal to noise ratio (SNR) as the random noise diminishes with the square root of the number of averaged images [3]. This improved SNR leads to a commensurate increase in the resolving power of the STM. The reason that this kind of multi-frame averaging (MFA) has so far not been performed routinely is that unique and locally varying distortions in each of the images prevent them from being aligned in perfect registry with each other.

In scanning probe microscopy (SPM), image distortions arise from thermal drift between the sample and tip, and from non-linearity of the piezo scanners [4, 5]. Image artefacts due to electrical interference such as mains noise (50 or 60 Hz) can also be a problem. Some mitigation strategies have been proposed such as reversed scans [4], orthogonal scans [5], or even spiral scans [6], but these are not in widespread use owing to the difficulty of their implementation. What is required then is an approach to maximise the utility of multiple scans with a fixed orientation using a software package that can correct image distortions, and hence perform automated MFA. An example of this type of software, called SmartAlign, is used for image processing in this paper and has been described in detail by Jones et al. [7]. Importantly, this software makes no assumptions about sample features or crystallography and requires no prior knowledge of the structure under investigation. Other codes also exist to perform affine-correction [8], or non-rigid registration of micrographs [9, 10]. However, these are variously not optimised for processing the multiple-scan data recorded here. These alternative approaches either do not incorporate the correction of non-linear STM distortions [8], require rapid scan-direction rotation [8], accept only pairs of perpendicular scans rather than many tens of frames [9], or do not make use of the serial-scanning prior knowledge [10]. These codes also do not include planar-ramp removal or integrated template matching, both of which are useful pre- and post-processing tools.

Here, we show how MFA can provide a step change in the resolving power of the STM, and how it has significantly changed the way we approach STM experiments. By knowing beforehand that we will perform MFA, we now acquire a sequential set of tens or hundreds of frames with the same imaging conditions (e.g. we keep the following parameters fixed: field of view, scan orientation and speed, sample bias, tunneling current, and feedback settings). In the past, we would have stopped acquiring images unless they were of higher quality than those previously captured. We demonstrate the power of the MFA approach with three examples to highlight separately the improvement of the fidelity, sensitivity, and selectivity in the STM data. The first example, imaging of the Si(111)-(7 × 7) reconstruction, is chosen because it is familiar to most users of ultrahigh vacuum (UHV) STMs and can, therefore, serve as an initial experiment for others to perform to repeat our results. For the Si(111) surface, we demonstrate a ~ sixfold improvement of the SNR ratio by averaging 41 raw images. The second example demonstrates a new result that would not have been observed without MFA. We are able to distinguish with sub-picometre precision the two different adsorption sites (fcc and hcp) of Ti atoms in a (2 × 2) honeycomb Ti_2_O_3_ monolayer on an Au(111) substrate. The final example shows how automated template matching can be used to distinguish chiral unit cells of the SrTiO_3_(111)-(4 × 4) reconstruction. This is only possible following MFA as the raw images are too noisy for chiral identification to be performed reliably.

## Results

### The Si(111)-(7 × 7) reconstruction

**Fig. 1:**
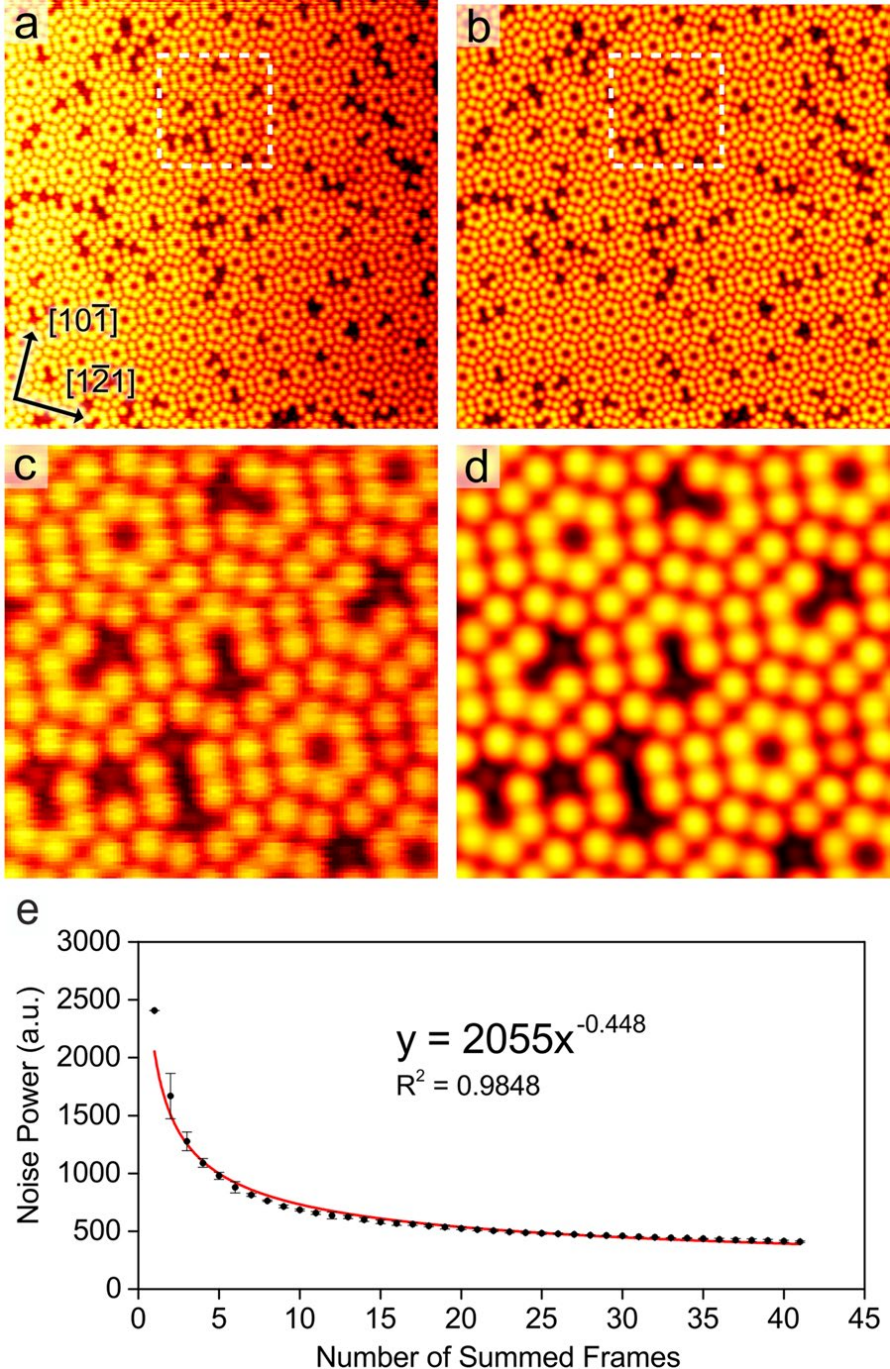
STM data from the Si(111)-(7 × 7) reconstruction. **a** Raw single frame (image width 33.2 nm, *V*_s_ = 1.3 V, *I*_t_ = 0.17 nA). **b** Image after ramp-subtraction, alignment, scan-correction and averaging of 41 sequential frames. **c** Magnified region from **a** (image width 8.3 nm). **d** Magnified region from **b**. **e** Fitting plot of noise power versus the number of summed frames. The plot shows the reduction in noise power with increasing number of averaged images

Si(111) samples were flashed in UHV to generate the familiar (7 × 7) reconstruction [11]. The surface was imaged in empty-states in the STM and sequential images were recorded with 33 nm image widths. One of these single images is shown in Fig. 1a, and represents a typical surface with a variety of atomic defects. The image in Fig. 1b is one where 41 scans of the same area have been averaged. To take full advantage of the MFA process, it is helpful to perform data up-sampling (interpolating over a finer image mesh) before MFA is carried out. The original STM images of 512 × 512 pixels were interpolated to 2048 × 2048 pixels. The improvement in the image noise can be more easily seen in the magnified frames (the bordered region in Fig. 1a, b) shown in Fig. 1c, d. It is obvious that the MFA image of Fig. 1d is less noisy than that of the raw image in Fig. 1c. We have quantified this reduction in noise by plotting the noise power against the number of averaged frames, as shown in Fig. 1e. The noise power was calculated by median filtering the images using a 3 × 3 kernel and subtracting these median filtered images from the originals. This process results in images containing only noise, and the standard deviation of the noise in these images is related to the noise power. As expected for a system that is mainly governed by shot noise, the reduction in the noise power can be approximated by an inverse-square law. The red line in Fig. 1e is the least squares fit to our data with an exponent of − 0.448, which can be compared to an exponent of − 0.5 if our data were to perfectly follow Poisson statistics.

**Fig. 2:**
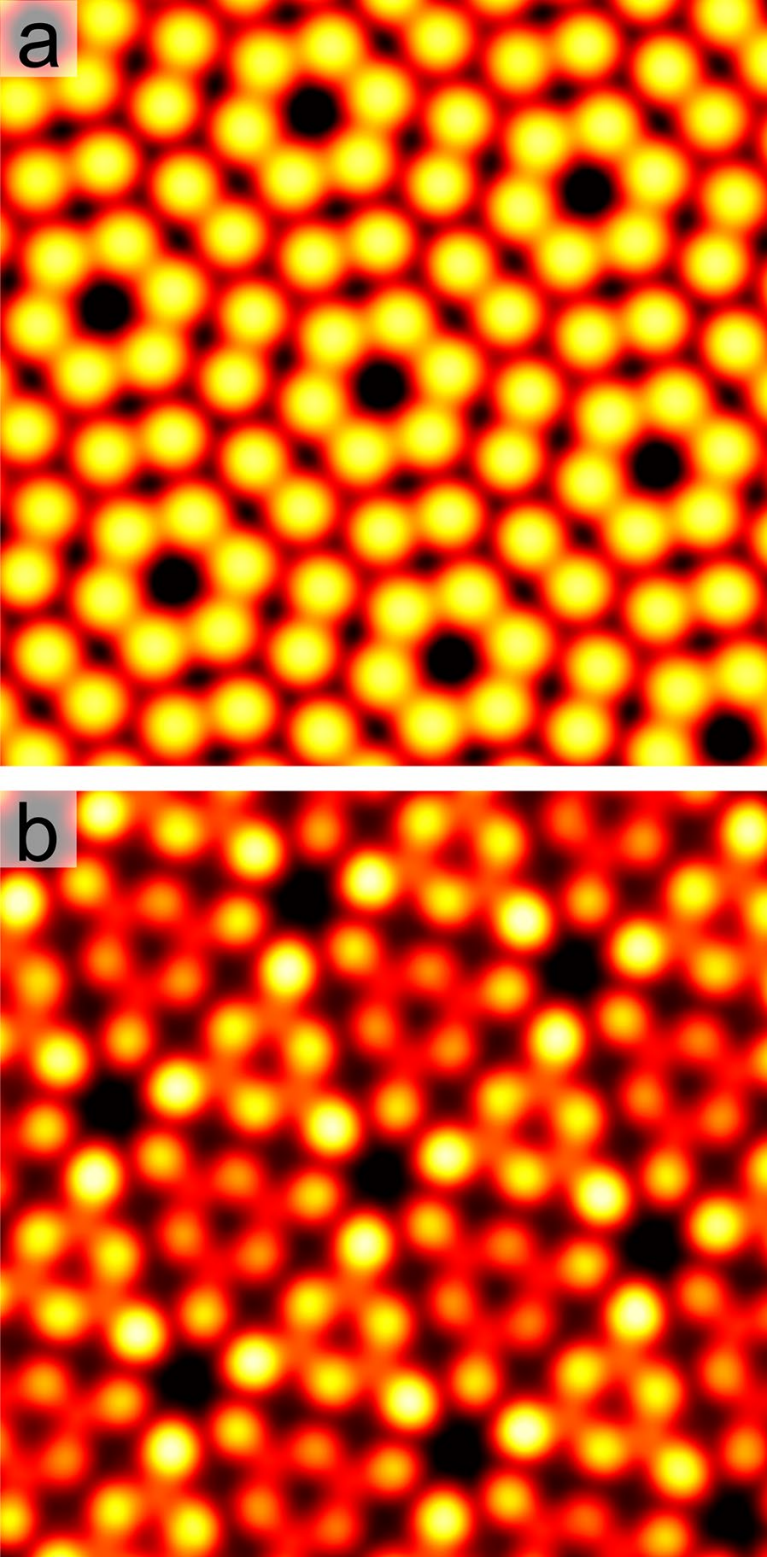
MFA images of defect-free regions of ~ 100 adatoms of the Si(111)-(7 × 7) reconstruction. **a** Empty-states image created from nine averaged tiles (image width 7.2 nm, *V*_s_ = 2.0 V, *I*_t_ = 0.7 nA). **b** Filled-states image from 27 averaged tiles (image width 7.2 nm, *V*_s_ = − 3.0 V, *I*_t_ = 1.0 nA)

A relatively common feature in STM software packages is a process called *unit cell averaging*. The user creates the surface unit cell grid in the STM image, and the software averages all of the cells to create an image with an enhanced SNR. This process has variable results, and tends to only work well on images where the image is already of high quality and has few distortions. We performed unit cell averaging over defect-free unit cells of the Si(111)-(7 × 7) reconstruction. This was achieved by selecting three separate crystallographically similar regions from a single STM image. Using the known threefold symmetry of the reconstruction [11], we rotated all the images twice by 120° and thereby tripled the number of tiles to average to nine. The result of MFA is shown in the empty-states image in Fig. 2a. We performed a similar procedure on filled-states images, but this time we selected three tiles each from three separate images and additionally rotated them all twice by 120°, thereby generating 27 tiles to average. The filled-states MFA result is shown in Fig. 2b. The images in Fig. 2 contain ~ 100 adatoms each and are comfortably superior to the traditionally unit cell averaged results where alignment is not as accurate.

Additional resolution enhancement of Si(111) images can also be achieved with particularly favourable tips [12]. Further SNR improvement is possible by exploiting known crystallographic relationships [13]. For the Si(111) surface, we could have additionally used the mirror symmetry of the (7 × 7) unit cell to further double the number of tiles that we averaged. For the high-quality images we used for Fig. 2 this mirror symmetry enhancement would have resulted in little additional benefit. However, in situations where the number of tiles is limited and the raw data are noisy, then full use of the crystallographic surface symmetry relationships should be made [14].

### Ti_2_O_3_ ultrathin films

**Fig. 3:**
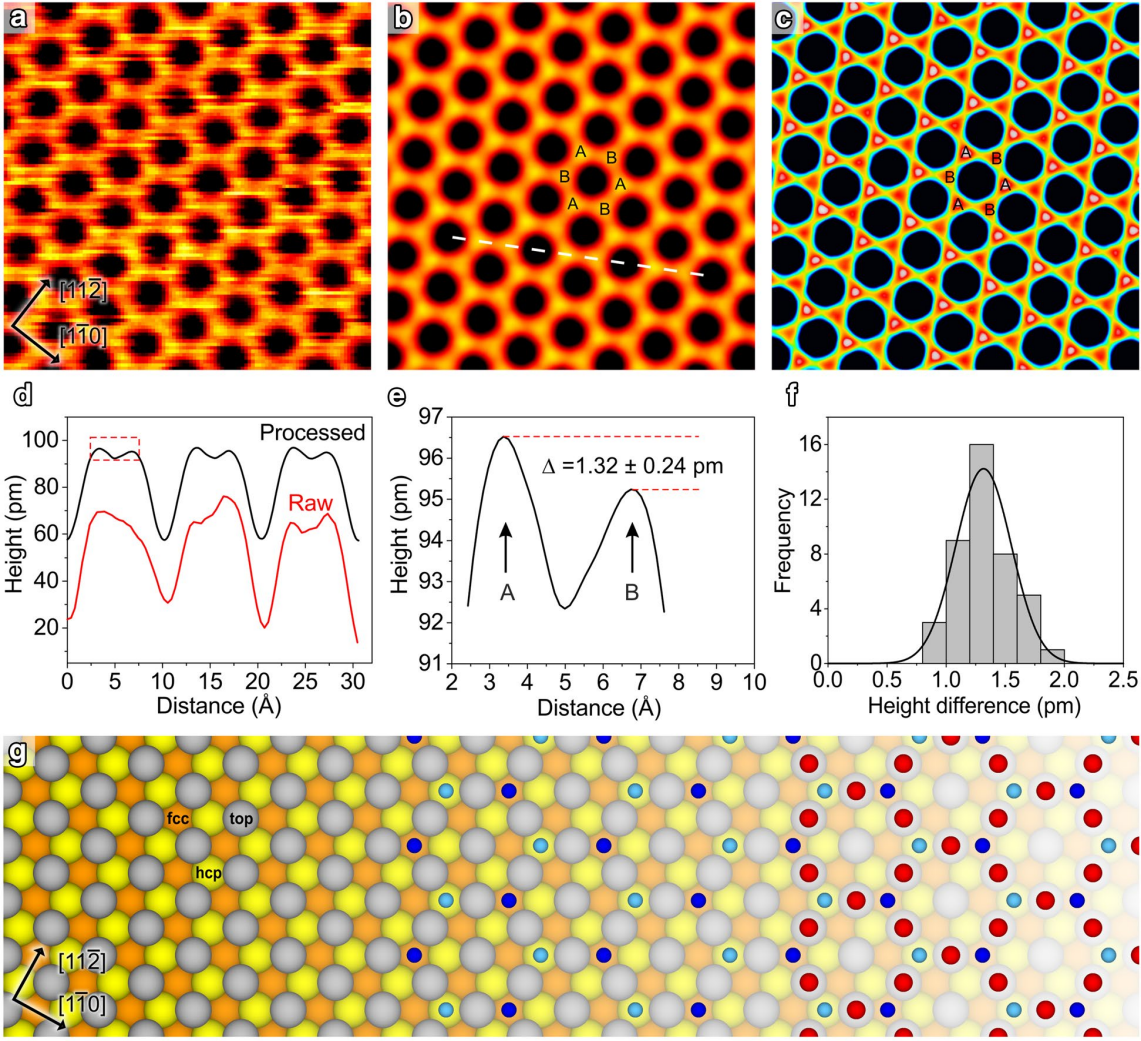
STM images and detailed analysis of the (2 × 2) honeycomb Ti_2_O_3_ ultrathin film on Au(111). **a** Raw STM image of the honeycomb structure (image width 4.2 nm, *V*_s_ = 1.0 V, *I*_t_ = 0.22). **b** Average of 528 tiles (image widths 4.2 nm, *V*_s_ = 1.0 V, *I*_t_ = 0.22–0.25 nA). The SNR is significantly improved and scan-distortions are corrected. Individual Ti atoms can be distinguished and are marked as A and B. **c** The same data as in **b** but with an exaggerated colour scale to highlight the small differences in peak height between A and B sites. **d** Line-profile from the raw and processed images from the dashed white line shown in **b**. The profiles are offset by 40 pm from each other for clarity. **e** Enlargement from **d** showing the 1.32 pm difference in height of the A and B sites. **f** Histogram of the height difference measured from 42 A–B pairs. **g** Model for the bare Au(111) surface (left part of figure) with hcp (yellow), fcc (orange), and top (grey) sites indicated. Ti atoms located on the fcc and hcp sites are in dark and light blue, respectively (centre of figure). O atoms are shown in red (right part of figure)

To demonstrate the sensitivity that can be achieved with MFA, STM images of honeycomb Ti_2_O_3_ ultrathin films grown epitaxially on Au(111) substrates were averaged [15]. Panels (a–c) of Fig. 3 show high-magnification empty-states STM images of the honeycomb Ti_2_O_3_(2 × 2) overlayer. To generate the MFA images 176 tiles of 4.2 × 4.2 nm^2^ defect-free areas were cropped from 88 raw frames of a continuous honeycomb overlayer. The 88 frames were recorded sequentially from the same area with no change in tip configuration and only minor changes in the STM imaging parameters. Figure 3a shows a typical raw image of one of the 176 tiles where the hexagonal rings can be identified, but where the image noise forms a significant barrier to seeing the fine atomic scale structure. The result of processing the 176 tiles, which were tripled to 528 tiles using the known threefold surface symmetry of this structure [15, 16] is shown in Fig. 3b. In this image the bright spots, corresponding to the Ti atoms [16], can now be clearly distinguished.

In Fig. 3c, the same data is shown as in Fig. 3b, but with an exaggerated colour scale. In this image, it can be seen that half of the Ti sites, marked as ‘A’ sites, are marginally brighter than the other half, marked as ‘B’ sites. This height difference can also be seen in the linescan indicated as a dashed white line in Fig. 3b and shown in Fig. 3d. The MFA processed image linescan (black) consistently shows a small difference in the height of the A sites and the B sites, whereas this difference is not apparent in the raw data (red) from a single tile. The height difference from the MFA processed data, highlighted in the dashed red box in Fig. 3d, is shown in Fig. 3e. Measurements of 42 A–B site pairs were taken (Fig. 3f) resulting in an average height difference of 1.32 ± 0.24 pm.

The origin of the asymmetry of the height of the A sites and B sites in the MFA images of Fig. 3b, c can be explained through an analysis of the structure of the Ti_2_O_3_ overlayer. Figure 3g is a diagram of the structure showing the bare Au(111)-(1 × 1) surface on the left, only the location of the Ti atoms shown in the centre, and the full Ti_2_O_3_ overlayer shown on the right. The Ti atoms (blue balls) are located in threefold hollow sites on the Au(111) surface, and the O atoms (red balls) are located in the outermost layer, on the ‘top’ sites. There are two types of threefold hollow sites, namely, hcp sites (yellow) and fcc sites (orange), as indicated in Fig. 3g. The structure of the overlayer results in half of the Ti atoms sitting in hcp sites, and the other half in fcc sites. Preliminary density functional theory calculations [17] show that the Ti atoms located on the hcp sites are marginally higher by less than a picometre than the ones on the fcc sites. It is according to this calculation that we assign the A and B sites to the fcc and hcp locations, respectively. These measurements show that through MFA we can perform atomic height determination to sensitivities comfortably better than a picometre.

### Chiral unit identification on SrTiO_3_(111)

**Fig. 4:**
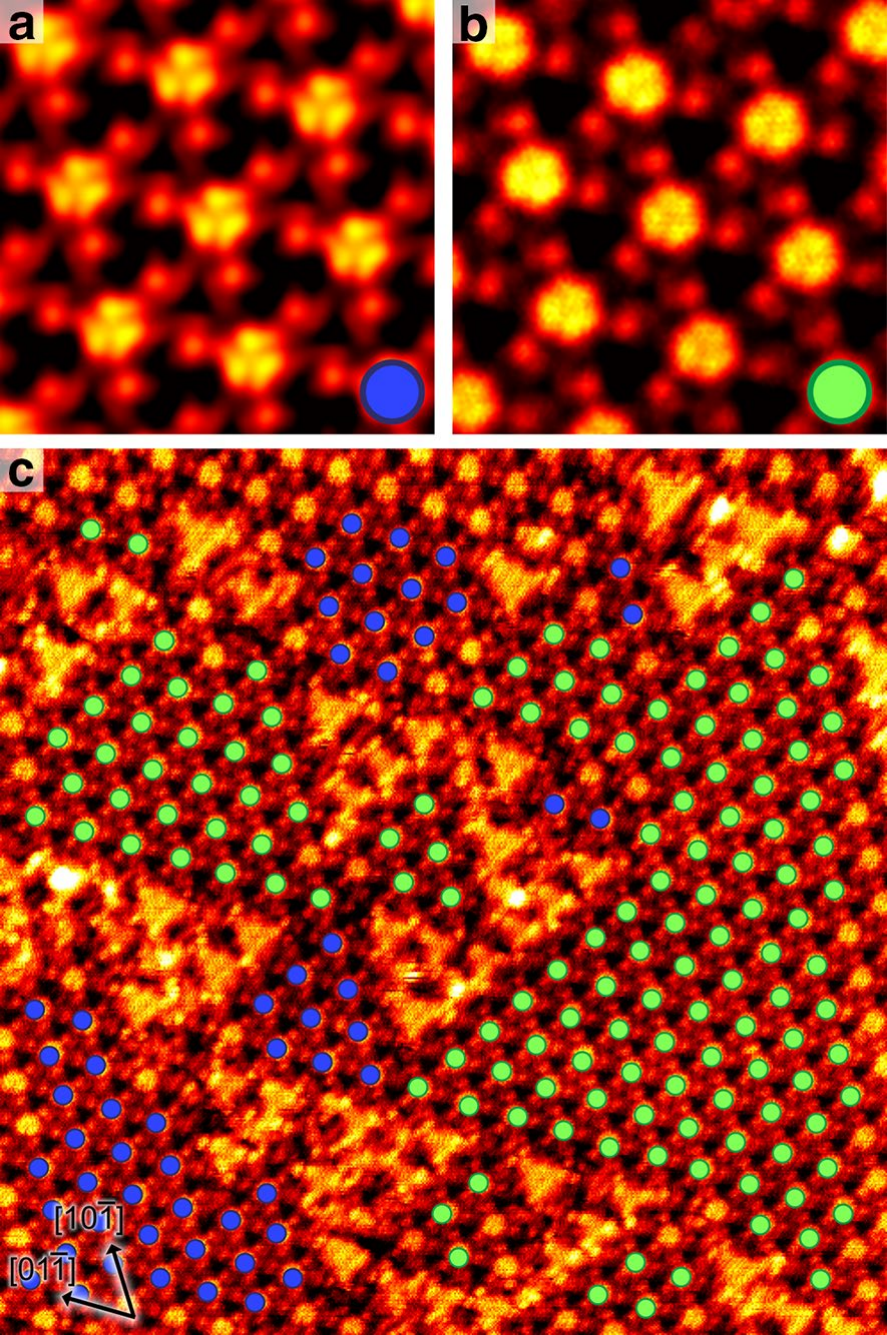
STM images of the SrTiO_3_(111)-(4 × 4) reconstruction. **a** Left-handed chiral units image constructed by averaging 297 defect-free tiles (image width 4.2 nm, *V*_s_ = 2.0 V, *I*_t_ = 0.5 nA). **b** Right-handed chiral units image constructed by averaging 15 defect-free tiles (image width 4.2 nm, *V*_s_ = 2.0–2.5 V, *I*_t_ = 0.5–0.65 nA). **c** Large area image where the reconstructed units associated with the left (blue) and right (green) chiralities have been automatically identified and labelled (image width 42 nm, *V*_s_ = 2.0 V, *I*_t_ = 0.5 nA)

STM is an invaluable tool for visualising chiral crystal terminations [18, 19] and molecular arrangements on surfaces [20–22]. Individual chiral units can sometimes be very challenging to identify accurately in raw STM data. Here, we demonstrate the utility of performing MFA to enhance the image SNR and thereby to be able to reliably automate the identification of chiral units. The STM images shown in Fig. 4 are of the SrTiO_3_(111)-(4 × 4) reconstruction. This is an unusual crystal termination in that the surface unit cells have chirality associated with them. The surface exhibits domains of each of the two types of unit cell that are non-superimposable mirror images of one another [18, 19], as shown in Fig. 4a, b. We assign left-handed chirality with a blue label to the units shown in Fig. 4a, and right-handed chirality with a green label to the units shown in Fig. 4b. Unintentionally, we were able to generate more high-resolution images that contained a majority of left-handed units, which is why the left-handed image (Fig. 4a) is constructed from 297 averaged defect-free tiles and has a better SNR than the right-handed image (Fig. 4b) that is constructed from only 15 tiles.

Owing to the similarity of the two types of unit cell, it can be challenging to distinguish chirality in raw noisy images. After applying a template-matching tool on an image that contained both chiralities, it was possible to automatically identify the left and right-handed units, as shown in Fig. 4c. It is immediately apparent that unit cells of the same chirality prefer to be located next to each other, which results in extended chiral domains, separated by disordered regions. It can also be seen in Fig. 4c that a few (4 × 4) unit cells have not been identified with either chirality. This occurs where there are local defects, or where the unit cell is near the image boundary and the template is too large to be entirely overlaid on the image.

The example of chiral recognition presented here uses a single crystal termination, but of course the same approach could readily be used for STM and AFM images of adsorbed chiral molecules. In our example, this automated procedure of the identification of repeating motif units is only possible on images that contain fine-structural detail. This emphasises the necessity for correcting for scanning artefacts and using subsequent image averaging to enhance the SNR. Other methods for automated feature extraction, such as those based on Fourier transforms [23], may also be used for chiral unit recognition.

## Discussion

Artefacts present in individual STM images can include affine-distortion (shear/stretch), non-linear scanning distortion, and abrupt image contrast changes resulting from structural tip changes. These artefacts arise due to thermal drift between the sample and the tip, piezo-scanner hysteresis, or from the laboratory environment, and are generally exacerbated as field-of-view or scanning time increase. Thus, the resolution of a single STM image is determined by the combination of imaging/instrumental artefacts and the intrinsic limit to resolution due to quantum mechanical interactions. In reality, it is the artefacts that limit the resolving power of most STMs. Imaging artefacts are often hidden by using filters such as median (real-space), Wiener and low-pass (Fourier-space) filters [24]. Nevertheless, median filters can distort the lattice or blur structural features. Wiener filters and low-pass filters involving Fourier transforms can introduce artificial periodicities or modify existing periodic features. A further point is that filters contain subjective elements where the researcher selects a specific filter to give the impression of resolution enhancement. However, we have shown in this paper that the MFA approach does not require any filtering steps. The scan-corrected images are reliable and highly reproducible due to the simple averaging process.

Previous studies from electron microscopy have shown that both the precision of image features (image intensity/feature-height) [7], and the lateral spatial precision of features [25], improves proportionally to the square root of the number of frames averaged. Here, we evaluate the signal–noise ratio (SNR) of aligned images for an increasing number of frames with similar findings. In the extreme case where many hundreds of frames are averaged, the median-filter and subtraction method becomes less sensitive to image noise and becomes limited by the finite sampling of the image data. This does not indicate that the precision is no longer improving, but merely that the resolvability of features or noise has become limited by pixel-size instead of SNR. In practice, the data in Fig. 1e shows that MFA of 30 images is sufficient for most applications because the rate of improvement diminishes significantly beyond this number.

The use of MFA techniques applied to STM images relies on obtaining images from the same area and under the same imaging conditions. Keeping sample bias and tunneling setpoint conditions the same is trivial; however the microscopist does not have control over the condition of the apical atom of the tip. Tip changes at the atomic level can result in significant changes in image contrast (e.g. Fig. 6 in Ref. [26]) and will often be the limiting factor that determines the number of sequential images that can be obtained. However, even as few as ten images will deliver a significantly improved SNR and it is common in STM to have this number of scans without a tip change.

We anticipate that MFA will find further application in STM images that suffer from serious distortions and thermal drift, such as those obtained during high-temperature imaging, and facilitate studies of surface diffusion [7]. Accurate alignment of images allows movies to be created that directly reveal dynamic behaviour without the distractions inherent in unprocessed sequences [27]. It is also possible to use automation to extract rare kinetic events that would be very time consuming to identify manually. The sensitivity of MFA may also provide new insights into the investigation of metal surfaces with corrugations less than 0.1 Å [24].

## Conclusions

With the three examples presented here, we have demonstrated the *fidelity*, *sensitivity*, and *selectivity* that can be achieved for STM data when using a multi-frame averaging (MFA) approach. This approach is made possible by the robust and automated non-rigid registration afforded by the SmartAlign software package. We demonstrate the approximately square-root relationship improvement in SNR upon image averaging, a sub-picometre precision height measurement, and the automated identification of chiral unit cells on a surface. These automated tools, which do not require prior knowledge of the surface structure, promise to facilitate more rapid and higher-precision studies of surfaces, making full use of the experimentalists recorded data sets. This advance allows a new study of surface pico-science to be developed where subtle variations in surface structure can now be seen, that hitherto were not detected because they were buried in noise. In future developments, it would be interesting to combine MFA with automated probe microscopy [28], to remove the substantial operator time commitment in obtaining hundreds of STM images of the same area.

## Methods

UHV-STM images were taken using two JEOL instruments, a JSTM4500s and a JSTM4500xt, both operating at a base pressure of 10^−8^ Pa. Constant current STM images were taken at room temperature using etched tungsten tips. The B-doped Si(111) samples were prepared by flashing at 1200 °C for 15 s to desorb the native oxide and cooled to room temperature to allow the (7 × 7) reconstruction to form. The (2 × 2) Ti_2_O_3_ honeycomb ultrathin films on Au(111) substrates were grown according to the description detailed in Ref. [15]. The SrTiO_3_(111)-(4 × 4) reconstructions were generated on 0.5 wt% Nb-doped samples according to the recipe described in Ref. [18].

To estimate the reduction in image noise power (and resulting improvement in image signal–noise ratio), images were 3 × 3 px median-filtered and subtracted from the originals to leave an estimate of the noise only. From these noise-estimates, the standard deviation was plotted as a metric of the noise power. This process was repeated for increasing numbers of averaged images leading to the inverse square-root trend behaviour. For very large numbers of frames being averaged, the SNR becomes so high that the median-filtering approach to estimation of noise power no longer holds and the improvement becomes harder to track. For the template-matching step, repeat-tile searching was performed using a thresholded cross-correlation approach. Local maxima in this cross-correlation indicate the positions of the repeats of the given motif which are then cropped out.

## Abbreviations

STM: scanning tunneling microscopy/microscope
SNR: signal to noise ratio
MFA: multi-frame averaging
AFM: atomic force microscopy/microscope
SPM: scanning probe microscopy
UHV: ultrahigh vacuum
hcp: hexagonal close packed
fcc: face-centered cubic
